# Effect of Ag Addition on the Gas-Sensing Properties of Nanostructured Resistive-Based Gas Sensors: An Overview

**DOI:** 10.3390/s21196454

**Published:** 2021-09-27

**Authors:** Sachin Navale, Mehrdad Shahbaz, Ali Mirzaei, Sang Sub Kim, Hyoun Woo Kim

**Affiliations:** 1 Division of Materials Science and Engineering, Hanyang University, Seoul 04763, Korea; stnavale2@yahoo.com; 2 The Research Institute of Industrial Science, Hanyang University, Seoul 04763, Korea; 3 Department of Materials Science and Engineering, Inha University, Incheon 22212, Korea; 4 Department of Materials Science and Engineering, Faculty of Engineering, Urmia University, Urmia 5756-151818, Iran; 5 Department of Materials Science and Engineering, Shiraz University of Technology, Shiraz 71557-13876, Iran; mirzaei@sutech.ac.ir

**Keywords:** Ag, decoration/loading, doping, gas sensor, sensing mechanism

## Abstract

Nanostructured semiconducting metal oxides (SMOs) are among the most popular sensing materials for integration into resistive-type gas sensors owing to their low costs and high sensing performances. SMOs can be decorated or doped with noble metals to further enhance their gas sensing properties. Ag is one of the cheapest noble metals, and it is extensively used in the decoration or doping of SMOs to boost the overall gas-sensing performances of SMOs. In this review, we discussed the impact of Ag addition on the gas-sensing properties of nanostructured resistive-based gas sensors. Ag-decorated or -doped SMOs often exhibit better responsivities/selectivities at low sensing temperatures and shorter response times than those of their pristine counterparts. Herein, the focus was on the detection mechanism of SMO-based gas sensors in the presence of Ag. This review can provide insights for research on SMO-based gas sensors.

## 1. Semiconducting Metal Oxide (SMO)-Based Gas Sensors

Toxic gases pose a major threat to modern life. Therefore, the early detection of toxic gases using reliable electronic gas sensors is extremely important. In this respect, chemiresistive gas sensors are potentially attractive due to their easy manufacturing, simple operating principle, and low cost [1,2,3,4,5,6,7,8,9,10]. In the area of resistive-based gas sensors, different semiconducting materials can be used for the realization of gas sensors. For example, the organic semiconducting materials such as organic π-conjugated materials have advantages of tailorable chemical structures, solution processability, and mechanical flexibility; thus, they are potential candidates for applications in low-cost, low-temperature, and portable gas sensors. Nonetheless, they have some shortages such as relatively poor sensitivity, slow response, and low recovery [1,2,3]. The functionalization of these materials with noble metals such as Ag through chemical methods can increase the response and selectivity to a particular gas; however, as far as we know, there is no or few studies in this regard. On the other hand, semiconducting metal oxides (SMO)-based gas sensors have advantages such as high response, high stability, relatively fast dynamics, simple fabrication, and low costs. So, they are very popular for the detection of various gases [4,5,6,7,8,9,10,11,12,13,14]. However, they work at high temperatures and show poor selectivity. 

**Figure 1:**
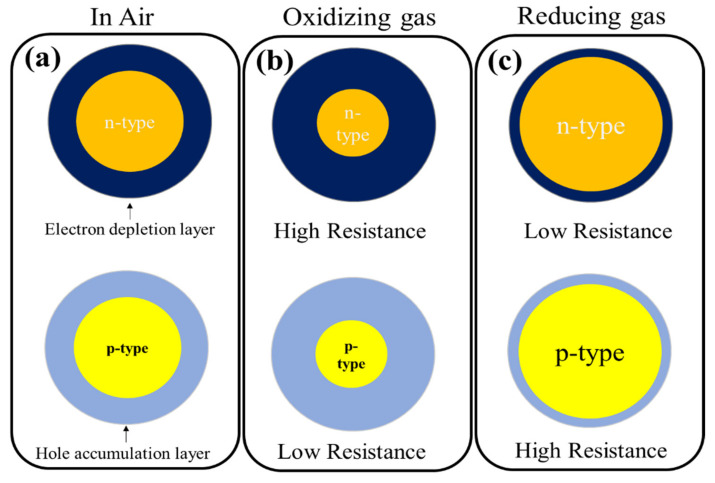
General gas-sensing mechanism of SMO-based gas sensors (**a**) n- and p-type SMO in air (**b**) in oxidizing gas atmosphere and (**c**) in reducing gas atmosphere [19,20].

Typically, in the laboratory, gas-sensing measurements are dynamically conducted in an enclosed test chamber of a defined volume equipped with an inlet and outlet for gas flow [15]. Generally, ideal gas sensors must be inexpensive, operate at low temperatures or room temperature, detect gases at low levels, and be highly stable, sensitive, selective, and fast [16]. Nano-based gas-sensing materials possess high surface areas and unique electrical properties and consequently are preferred over their micron-sized counterparts for the development of gas sensors [17]. The detection mechanism of SMOs significantly depends on the modulation of electrical resistance due to the interaction of the sensing layer with the target gases [18]. The general sensing mechanism of resistive-based gas sensors is schematically shown in Figure 1 for n-type and p-type SMOs, in the presence of oxidizing and reducing gases. When an SMO is exposed to air, depending on the sensing temperature, the adsorption of oxygen on the sensor surface causes the ionization of oxygen molecules in the form of molecular or atomic ions. As a result of oxygen adsorption, a so-called electron depletion layer with a low concentration of electrons forms on the surfaces of n-type SMOs, which has a higher resistance than the core region of the metal oxide. Alternatively, a so-called hole accumulation layer forms on the surfaces of p-type SMOs, which has a lower resistance than the core regions of the metal oxides due to an increase in the number of holes as major carriers (Figure 1a). Upon exposure of the gas sensor to reducing gases, the gases will be adsorbed on the surface of the sensing layer and react with the already adsorbed oxygen ions, liberating the electrons back to the surface of the gas sensor. Thus, the width of the electron depletion layer in n-type metal oxides decreases, leading to a decrease in the resistance of the sensor. For p-type metal oxides, the width of the hole accumulation layer decreases because of the combination of the released electrons with holes, resulting in an increase in sensor resistance, which contributes to the sensor signal (Figure 1b). For oxidizing gases due to the further abstraction of electrons, the widths of both the electron depletion layer and hole accumulation layer increase, leading to an increase in resistance in n-type metal oxides and decrease in the resistance in p-type metal oxides (Figure 1c) [19,20].

Since the adsorption of gas molecules on the surface of the gas sensor depends on the surface area, different morphologies have been investigated for gas-sensing applications. In fact, the larger surface area results in more availability of the adsorption sites on the surface of the gas sensor, resulting in a higher response of the gas sensor [21]. In addition to the increase in the surface area, several strategies, such as doping [22], decoration of noble metals [23], and formation of heterojunctions [24] have been reported to enhance the gas-sensing performances of SMOs. Selectivity is a challenging issue of resistive-based gas sensors and is not completely solved for gas sensors. However, there are some strategies to increase the selectivity of the gas sensor to a particular gas. For example, the control of sensing temperature, use of membranes, use of catalytic materials, and noble metal decoration have been suggested to increase the selectivity of resistive-based gas sensors [25]. 

## 2. Noble Metal Decoration

In the area of SMOs-based gas sensors, there are many review papers [26,27,28,29,30,31,32,33,34,35]. However, less attention has been paid to review the effect of noble metals on the gas-sensing performance of SMO-based gas sensors. Mirzaei et al. [36], Korotcenkov et al. [37], and Luo et al. [38] have discussed the effect of Pd, Au, and metal doping, respectively, on the gas response of SMO-based gas sensors. Mirzaei et al. [23] briefly discussed the effect noble metals on the gas response of SMO-based gas sensors. So, as far as we know, there is no review paper devoted solely to the effect of the addition of Ag in the form of decoration and doping on the gas response characteristics of SMO-based gas sensors. Thus, in this review paper, we have tried to cover this area of SMO-based gas sensors. It should be noted that in this manuscript, both decoration and loading are interchangeable words, and both of them mean the dispersion of Ag NPs on the surface of SMO.

**Figure 2:**
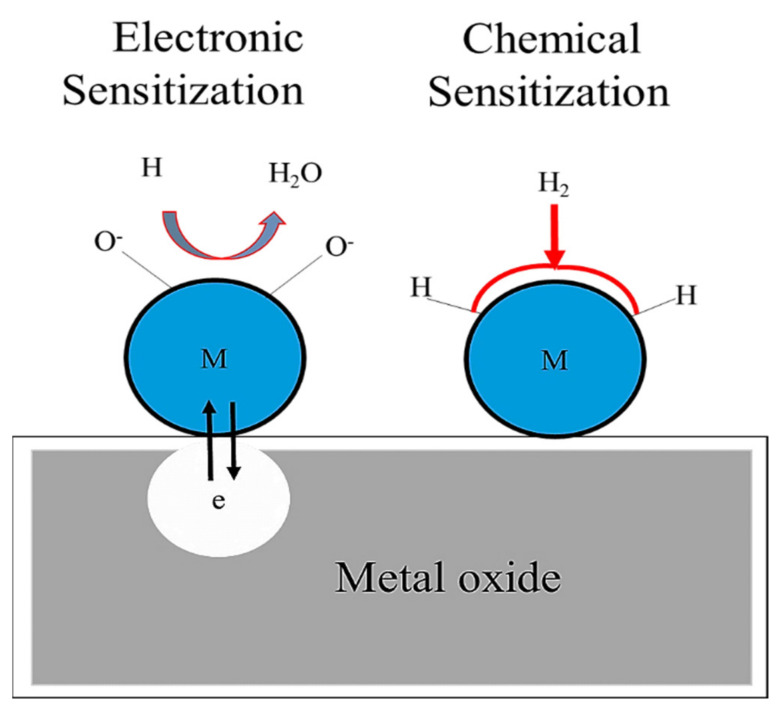
Schematic of chemical and electronic sensitizations in noble metal-decorated SMOs (in the figure, M = Metal, e = electron, O^−^ = adsorbed oxygen) [22,31].

Among the various strategies, the decoration of SMOs with noble metals is perhaps the most attractive strategy, and enhanced gas responses of the resulting sensors are mainly attributed to the chemical and electronic sensitization effects of noble metals [23,39]. Typically, noble metals are recognized for their catalytic activities, and they can decrease the adsorption activation energies of gases on the surfaces of SMOs and enhance the sensing activities of SMO-based sensors. The surface doping of noble metals may generate active surface sites that facilitate the preferred adsorption of target gases and increase their concentration. Moreover, noble metals can offer reaction paths that can reduce the activation energy and then improve the reaction rate and selectivity, sensitivity or response, and reliability of the sensor [22,23]. Generally, the chemical sensitization effect originates from a spillover effect, where the noble metal may act as a potential site for the adsorption and dissociation of O_2_ molecules [40]. Subsequent spillover onto neighboring SMOs results in enhancement of the adsorption rate of O_2_. Additionally, noble metals may catalyze the dissociation of target gases and boost the rates of reactions between the target gases and the previously chemisorbed O_2_ species. Basically, the chemical sensitization effect particularly depends on the operating temperature at which the catalytic pathway is preferred and is definite to the case where chemical affinities exist among the species concerned. However, the electronic sensitization effect arises from the direct electronic interaction between the promoter and the semiconductor surface. Typically, the electronic sensitization effect depends on the presence of a junction potential barrier between the semiconductor and the noble metal. Differences between the Fermi levels of the SMO and the catalyst cause the depletion or accumulation of charge carriers at the semiconductor, adjacent to the particles, because of the pinning effect [41,42,43]. When the oxidation state of a noble metal, including Ag, changes with respect to the surrounding atmosphere, the electronic state of the semiconductor accordingly varies because Ag can form Ag_2_O or AgO in air, whereas Ag_2_O or AgO is simply reduced to metallic Ag when exposed to a reducing gas. In fact, Ag_2_O or AgO create an electron-depletion layers within the semiconductor, whereas the electronic interaction is disrupted when Ag_2_O or AgO is reduced to Ag [41,42,43]. As an example, a schematic of the chemical and electronic sensitization effect of noble metals and SMOs (such as TiO_2_) with H_2_ gas as the target gas is shown in Figure 2 [22,42]. 

In this case, the H_2_ molecules may dissociate into H atoms on noble metal clusters and then spillover onto the TiO_2_ surface, which accelerates the reaction [22,39,42]. Herein, the noble metal clusters simply reduce the activation energy of the reaction or increase the reaction rate. The electronic sensitization effect can be attributed to electronic interactions between TiO_2_ and noble metal clusters. Accordingly, because of the difference between the work functions and electron affinities of TiO_2_ and noble metal, a depleted space charge region is generated close to the noble metal/TiO_2_ interface, which causes band bending [23,39]. The electronic sensitization effect is primarily controlled by the spillover effect of H_2_ on the TiO_2_ surface and reduces the TiO_2_ resistance via the transfer of electrons at the noble metal/TiO_2_ interface. In another case of doping, the dopant atoms enter the TiO_2_ framework and subsequently distort the TiO_2_ lattice, thereby producing many oxygen defects that provide higher local electric fields and facilitate the dissociation of H_2_ [22]. Consequently, the rate of the reaction between H and chemisorbed oxygen ions is enhanced, thereby reducing the response time. Additionally, more oxygen species may adsorb around the doped atoms than those in the case of pristine TiO_2_, thereby increasing the resistance and sensitivity or response of the sensor [23,39,41,42].

Generally, noble metals are very expensive. If even small amounts of noble metals are used for decorating or doping SMOs, the overall cost of the sensor inevitably increases. Nevertheless, among various noble metals such as Pd, Pt, and Au, Ag has the lowest price. Furthermore, Ag has several interesting properties, for example, high efficiency and high reactivity toward O_2_ adsorption. Additionally, the Ag ion has been reported as a high mobility cation, which has been utilized in resistive-switching devices [43,44]. Moreover, Ag catalysts are efficient for not only improving the sensitivity of the sensor but also for reducing the working temperature owing to the unique catalytic and electronic characteristics of Ag. As an effective catalyst, Ag can attract O_2_ molecules in the air, transfer them to the surfaces of SMOs, and subsequently promote the capture of electrons from the conduction band of SMOs by these O_2_ molecules [41,42,43,44]. Therefore, Ag can be an appropriate sensitizer for introduction into the surfaces of SMO-based resistive gas sensors. Although the addition of Ag may boost the performance of the sensing material, a reasonable doping level/concentration of Ag is needed to realize the maximum sensing response. Typically, the decoration of SMO with Ag at an optimal concentration can result in the highest sensing performance of SMO. In fact, as the catalytic and electronic effects of Ag are unsatisfactory, the best sensing properties cannot be achieved when the loading amount of Ag is extremely low. When the loading or decoration concentration of Ag is substantially high, the electrons are conducted along metallic Ag, thereby decreasing the response of the gas sensor [45]. Generally, the best outcomes are obtained using ultrafine Ag nanoparticles (NPs) at low concentrations (0.1–10 wt.%) and high surface dispersion, promoting the catalytic activity of the sensor without compromising the function of the sensing layer [46]. In the following section, we discuss some of the most recent and important studies on the application of Ag-decorated/loaded semiconductors for the detection of toxic and hazardous gases.

## 3. Ag-Decorated/Loaded Gas Sensors

### 3.1. Ag-Decorated/Loaded Acetone (CH_3_COCH_3_) Gas Sensors

**Figure 3:**
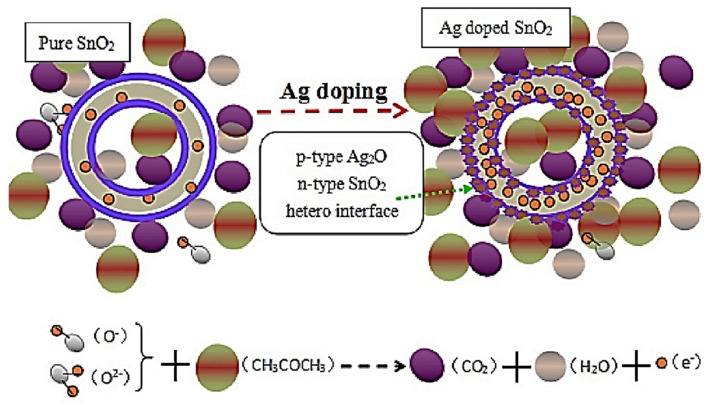
Schematic representing the CH_3_COCH_3_ sensing mechanism of pure and Ag-doped SnO_2_. Reprinted from reference [48] with permission from Elsevier.

CH_3_COCH_3_ belongs to the family of volatile organic compounds (VOCs) that are employed as solvents in various industries. CH_3_COCH_3_ at higher levels may have a negative impact on the central nervous system and may be harmful to the eyes and nose. Therefore, the occupational threshold limit value for CH_3_COCH_3_ was set at 250 ppm considering a time-weighted average of 8 h [47]. Accordingly, the development of sensitive CH_3_COCH_3_-sensing devices is required from the perspective of safety. In this regard, some attempts have been made to establish selective CH_3_COCH_3_ sensors by adding Ag into SMOs. For instance, Xu et al. [48] described the synthesis of Ag-decorated SnO_2_ hollow nanofibers (NFs) with large surface areas and their CH_3_COCH_3_ sensing properties. Typically, noble metals have higher electrical conductivities that enable the rapid transfer of electrons and catalysis of the oxidation of reducing gas molecules such as VOCs [45,46,47,48]. Thus, noble metal-incorporated sensing materials can be suitable candidates for detecting VOCs. The developed Ag-decorated SnO_2_ sensor showed an excellent response and higher selectivity for CH_3_COCH_3_ at 160 °C. A schematic of the sensing interactions between the CH_3_COCH_3_ gas molecules and pure and Ag-decorated SnO_2_ is shown in Figure 3. Owing to the presence of p-type Ag_2_O crystals, p-n heterojunction interfaces formed between p-type Ag_2_O crystals and n-type SnO_2_, generating a broader depletion layer on the SnO_2_ side, thereby increasing the initial resistance of the sensing material. Upon exposure to CH_3_COCH_3_, significant modulation of the heterojunctions occurred and contributed to the sensor response. The Ag NPs produced Ag_2_O in air, which has facilitated the development of highly electron-depleted layers while removing electrons from SnO_2_. This process has increased the total resistance and the area adjacent to the Ag–SnO_2_ interfaces, thus increasing the sensitivity of SnO_2_ to CH_3_COCH_3_. Furthermore, one-dimensional (1D) SnO_2_ hollow nanostructures fabricated via electrospinning considerably facilitated the diffusion and transport of electrons in CH_3_COCH_3_ molecules, thereby rapidly and substantially changing the sensor resistance. Additionally, SnO_2_ hollow NFs with both outer and inner surfaces exhibited high surface areas and eventually contributed to high CH_3_COCH_3_ responses. In summary, the significantly high CH_3_COCH_3_ sensing performances of Ag/SnO_2_ composites are attributed to the Ag dopant and 1D NF structures of SnO_2_. Ag with high electrical conductivity enabled the rapid transfer of electrons and catalyzed the oxidation of the reducing CH_3_COCH_3_ gas molecules. Moreover, the SnO_2_ NFs with higher surface areas led to an effective dispersion of catalyst particles and possessed a porous tube-like structure that promoted rapid gas flow and a superior ability to store and release oxygen ions.

In another study, Kilic et al. [49] reported the CH_3_COCH_3_ sensing characteristics of Ag-loaded TiO_2_ nanorods (NRs). They used a seed-mediated hydrothermal approach to grow TiO_2_ NRs and subsequently loaded Ag onto TiO_2_ NRs by thermally evaporating metallic Ag at different times of 30, 45, and 90 s. The authors demonstrated that the enhancement in the CH_3_COCH_3_ detection performance of the Ag-loaded TiO_2_ (45 s) sensor was due to the catalytic activity of Ag. Actually, Ag loading increased the number of adsorption sites on the surface of TiO_2_ and accelerated the rate of electron exchange between the TiO_2_ surface and CH_3_COCH_3_ molecules. Under a CH_3_COCH_3_ atmosphere, the bonds between CH_3_COCH_3_ molecules were easily dissociated by Ag, allowing these molecules to quickly interact with the chemisorbed oxygen species. Moreover, the decoration of Ag NPs onto TiO_2_ NRs pinned the Fermi level of TiO_2_ because of the transfer of electrons from TiO_2_ to Ag. This led to surface band bending and induced a more pronounced electron–hole separation effect, thereby enhancing the sensitivity of the sensor to CH_3_COCH_3_. The lower CH_3_COCH_3_ response of the Ag-loaded TiO_2_ (90 s) sensor was associated with the accumulation of Ag clusters on the TiO_2_ surface, which impeded O_2_ diffusion within TiO_2_ and reduced the catalytic activity of Ag.

### 3.2. Ag-Decorated/Loaded Chlorine (Cl_2_) Gas Sensors

**Figure 4:**
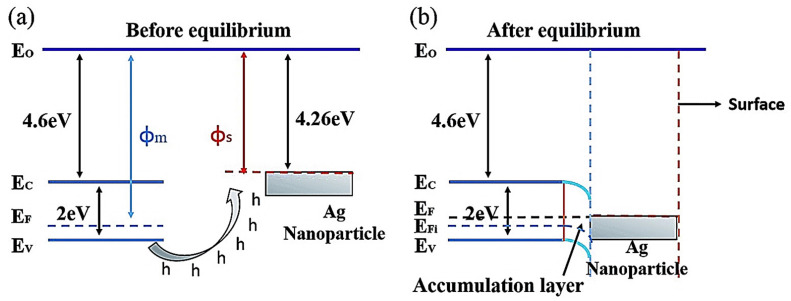
Energy band diagram and hole transfer in Ag NP-decorated BFO NSs (**a**) before and (**b**) after equilibrium [50]. Reproduced from https://pubs.rsc.org/en/content/articlelanding/2018/ra/c8ra06247a (accessed on 26 September 2018) from RSC. (This article is licensed under a Creative Commons Attribution-NonCommercial 3.0 Unported Licence).

Cl_2_ gas is very hazardous to the human respiratory mucous membrane at concentrations in the range 0.2–3.5 ppm. It causes psychological disorders, skin infections, and even liver damage. Hence, the early detection and successive monitoring of hazardous Cl_2_ by reliable gas sensors are highly important. Few studies have been reported on the Cl_2_ detection properties of Ag-loaded sensors. Li et al. [50] illustrated the effect of Ag loading on the Cl_2_ response of bismuth ferrite (BiFeO_3_, BFO) nanospheres (BFO NSs); they synthesized BFO NSs using a sol–gel route and successively loaded Ag NP onto BFO NSs via a photodeposition technique. Typically, BFO has a distinct surface reactivity, O_2_ adsorption ability, and a narrow band gap, which are beneficial for improving the gas-sensing characteristics. Therefore, BFO NSs are important candidates for the detection of Cl_2_. The observed response of the optimum 4 mg AgNO_3_-modified BFO sensor to 10 ppm Cl_2_ was 72.62 at a working temperature of 240 °C. This response was 2.5 times that of the pure BFO sensor. A schematic of the energy band structure and hole transfer after the loading of Ag NPs is shown in Figure 4. Upon the loading of Ag NPs onto BFO, the positive charges on BFO reduced, and downward band bending occurred because the holes on the BFO NSs transferred to the Ag NPs. Along with the separation of electrons and holes, the electrons probably combined with O_2_ and Cl_2_ on the surface of the gas sensor. No barriers were formed because of the reduction in the number of conduction band electrons, thus supporting the progress of the reaction. Moreover, the Fermi levels of Ag and BFO correspond to one another at the same level, following contact because BFO (Φm) has a lower work function than that of Ag (Φs). Here, Ag NPs might also be used as distinct adsorption sites for O_2_ and Cl_2_. Furthermore, at the Ag/BFO interface, a Schottky junction was created that decreased the intergranular barriers and enhanced the interfacial effect [50]. 

In contrast, the number of electrons at the reaction sites increased, and the reaction quickly saturated because of the rapid transfer of charge carriers from BFO to Ag. Consequently, the gas response significantly improved, and the response time shortened. However, the amount of adsorbed O ions decreased when high amounts of Ag were loaded onto the BFO NSs, which reduced the reactions of O_2_ and Cl_2_, thereby decreasing the sensor response. Thus, this study demonstrates that the addition of noble metals presents a new way of enhancing the sensitivity of p-type semiconductors toward Cl_2_ gas.

### 3.3. Ag-Decorated/Loaded Acetylene (C_2_H_2_) Gas Sensors

**Figure 5:**
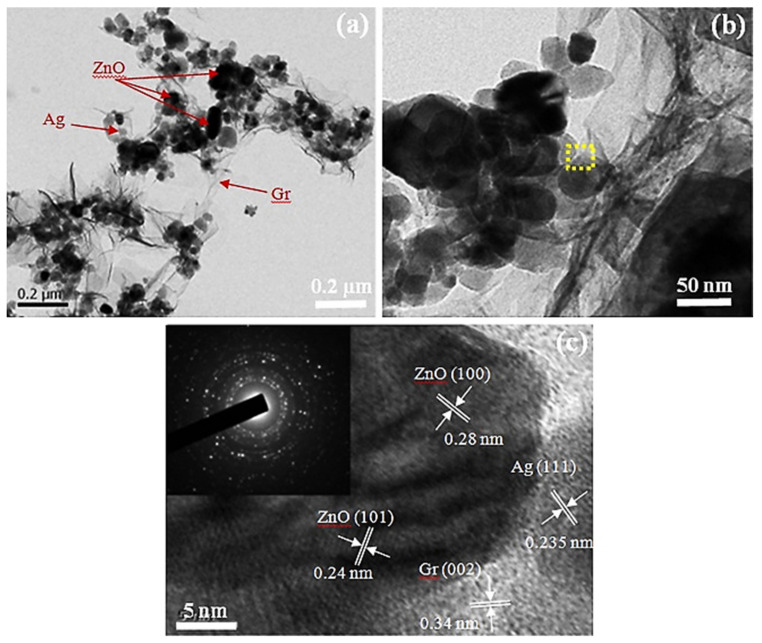
(**a**) TEM and (**b**,**c**) HRTEM images of the ZG–Ag3 hybrid (inset shows the equivalent SAED pattern). Reprinted from reference [51] with permission from Elsevier.

C_2_H_2_ is a flammable and colorless gas with a peculiar odor and is commonly utilized as a fuel in oxyacetylene welding and metal cutting and as a raw material in various industries. C_2_H_2_ poses serious threats due to its inherent instability, primarily in the cases of liquefaction, pressurization, heating, or mixing with air. This means that C_2_H_2_ can cause massive explosions if it leaks. Accordingly, from environmental and safety perspectives, the development of highly efficient C_2_H_2_ sensors is becoming increasingly important [31,51,52]. In this regard, Uddin et al. [51] described the C_2_H_2_ sensing properties of chemically synthesized 0–5 wt.% Ag-loaded ZnO-reduced graphene oxide (ZG-Ag) ternary hybrids. Generally, herein, the use of rGO was preferred over that of graphene because of difficulties in the large-scale production of graphene and lack of functional groups and band gaps in graphene [53]. Transmission electron microscopy (TEM) and high-resolution TEM (HRTEM) images (Figure 5) support the Ag/ZnO/Gr configuration of these heterostructures.

The Ag-loaded sensor based on ternary hybrids showed a higher response than that of the sensor without Ag. The sensor without Ag could detect 16–100 ppm C_2_H_2_ at 250 °C. The response of the 3 wt.% Ag-loaded ZnO–Gr (ZG-Ag3) sensor was 22 at 150 °C, indicating that 3 wt.% was the optimum loading amount of Ag. The loading of 3 wt.% Ag onto the ZnO–Gr sensor not only enhanced the sensor response but also reduced the optimum sensing temperature. Ag has a superior capacity to dissociate O_2_ than ZnO, and it catalyzes the dissociation of molecular O_2_ on the sensor surface, which generates higher C_2_H_2_-sensing active sites and thereby a higher sensor response. The development of depletion layers around the ZnO NPs due to the presence of Ag was mostly correlated with the modulation of nano-Schottky barriers, thereby improving the low-temperature surface reactivity. Upon the loading of Ag onto the ZnO–Gr sensor, further dynamic sites generated at the sensing layer/Ag interfaces because of the spillover effect, and a larger number of C_2_H_2_ molecules adsorbed on the sensor surface. Therefore, a high sensor response was obtained when the loading amount of Ag was optimal. Nevertheless, when an excess amount of Ag was loaded, the O_2_ molecules dissociated on the surface and surpassed the percolation threshold, which caused an overlapping spillover zone, ultimately affecting the efficient transfer of O_2_ and decreasing the chances of C_2_H_2_ adsorption; this reduced the sensor response.

**Figure 6:**
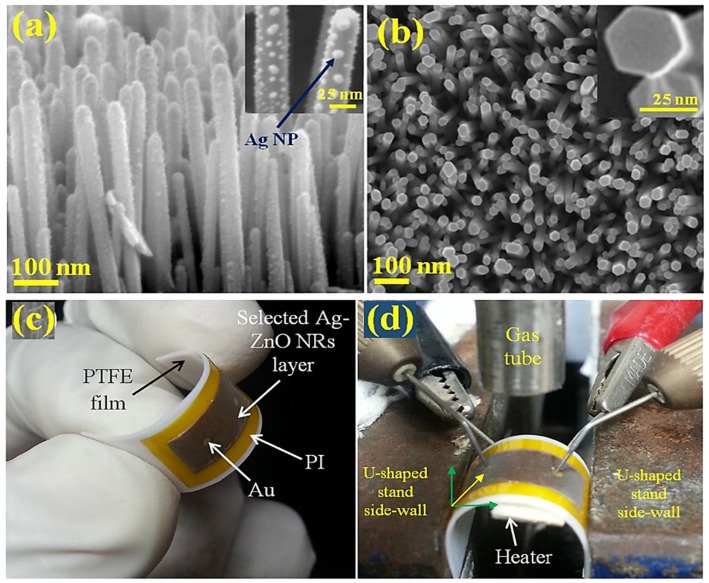
Images of (**a**) cross-section and (**b**) in-plane view of 8 s Ag-loaded ZnO NRs, (**c**) the Ag-decorated ZnO flexible gas sensor, and (**d**) the experimental setup at one bending angle. Reprinted from reference [54] with permission from Elsevier.

In another study associated with the detection of C_2_H_2_, Uddin et al. [54] designed a C_2_H_2_ sensor using Ag-decorated ZnO NRs supported by a flexible polyimide (PI)/polytetrafluoroethylene (PTFE) substrate (Figure 6). 

Flexible gas sensors should have the following characteristics: (i) the ability to detect gases at low concentrations and (ii) low- or room-temperature operation [55]. In this study, radio frequency magnetron sputtering (125 W, 7 mTorr) was applied to load Ag NPs onto the sensing layer of ZnO NRs in an Ar environment at different loading times of 6, 8, and 10 s. Gas-sensing analysis demonstrated that the flexible sensor developed using 8 s Ag-loaded ZnO NRs showed a higher response of 13.8 toward 100 ppm C_2_H_2_ at 200 °C than those of the pristine (3.21), 6 s (9.33), and 10 s (9.57) Ag-loaded sensors. Furthermore, upon repeated bending and relaxation for up to 5 × 10^4^ cycles, the sensor response only slightly decreased, which demonstrated the outstanding sustainability and mechanical strength of the designed flexible sensor. This performance improvement was attributed to the integration of the Ag–ZnO structure with the flexible PI substrate, which improved the physical binding and adherence of the Ag–ZnO structure to the supporting substrate. ZnO NRs with small diameters ensured an improved surface area and a larger aspect ratio of the resulting sensor. This caused inadequate surface atomic coordination and higher surface energy, leading to better adsorption of O ions and hence improved sensor response. Furthermore, because of catalytic interactions, the Ag NP/ZnO NR interface might produce further charge carriers or O vacancies on the sensor surface. In the case of Ag-loaded ZnO NRs, the ZnO surface transformed from an electron-depletion state to an almost flat band state along with a redox change at Ag. Additionally, the improved C_2_H_2_ sensing activity observed at an optimum temperature of 200 °C was ascribed to the height of the Schottky barrier between the ZnO grains and Ag in the Ag-loaded ZnO NRs. The improvement in the performance of the Ag-loaded ZnO NRs may also be controlled by changing the Ag concentration for the sensitive variation of the Ag oxidation state during exposure to C_2_H_2_. When the Ag loading amount was high, a thick Ag layer developed on the ZnO NR surface, which decreased the efficacy of the open surface porosity of ZnO, thereby reducing the gas sensor response. Herein, the observed higher selectivity of the Ag/ZnO NR sensor toward C_2_H_2_ was attributed to the chemical sensitization effect. Since Ag has excellent catalytic activity, it acts as a certain adsorption site for the dissociation of O_2_ and separation of H_2_ molecules owing to the spillover effect.

### 3.4. Ag-Decorated/Loaded Triethylamine (TEA) Gas Sensors

TEA, a clear and flammable liquid with a strong odor of ammonia, is widely used in chemical industries [56]. However, TEA can cause many severe health issues, such as pulmonary edema, gastroenteritis, headaches, and even death, because of its toxicity [57]. Additionally, dead fish and other decaying marine products may release TEA, and the concentration of TEA is expected to progressively increase over time [58]. Consequently, TEA may serve as a chemical indicator to assess the quality of marine food [59]. Therefore, designing an advanced sensor for the detection and monitoring of TEA is required. In contrast, recently, three-dimensional (3D) structural nanomaterials have received considerable attention in gas-sensing applications. In fact, 3D structures may offer highly efficient specific surface areas that improve the response of the corresponding gas sensor. Accordingly, Shen et al. [60] reported the TEA sensing characteristics of Ag-loaded 3D porous ZnO microspheres. Their morphological studies indicated that the ZnO microspheres (3–5 μm in size) were assembled by numerous thin and porous nanosheets with sizes of ≈20 nm. The developed sensor exhibited better cross-selectivity toward TEA, which was significantly attributed to the differences between the reactivity of the target gases caused by their different bond energies and chemical molecular structures. The reported C-N, C-O, C-H, C=C, C=O, and O-H bond energies are 307, 326, 414, 610.3, 798.9, and 458.8 kJ/mol, respectively. As a result of the lower C-N bond energy, the TEA molecules were more easily reduced by Ag–ZnO. 

**Figure 7:**
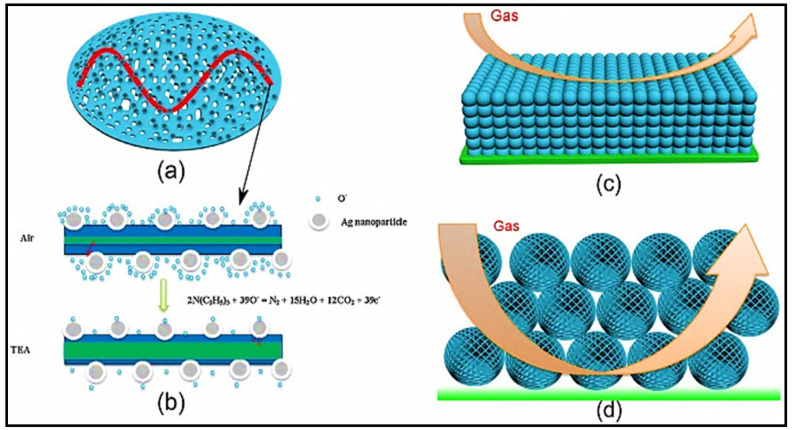
Schematic of the (**a**,**b**) TEA gas-sensing mechanism of the Ag–ZnO sensor and (**c**,**d**) structures of the microspheres with the illustration of large pores. Reprinted from reference [60] with permission from Elsevier.

In this case, the baseline resistance of the Ag-loaded sensor (2737.8 MΩ) was higher than that of the pristine sensor (28.5 MΩ). Furthermore, O species may more readily adsorb on the Ag NPs via a known spillover effect. Thus, highly active negative O ions spilled on the ZnO surface, which extracted the electrons from the conduction band of ZnO, thereby increasing the thickness of the electron-depletion layer and consequently the sensor resistance. Furthermore, the active O species accelerated the reaction of the sensor with TEA. Overall, the widely dispersed Ag NPs were highly favorable to the spillover effect and might catalyze the sensing reaction to increase the response of the sensor to TEA (Figure 7a,b). Finally, the special hierarchical 3D porous nanostructure comprised hundreds of porous nanosheets was an additional meaningful component for the excellent TEA sensing properties of the Ag-loaded gas sensor. This unique structure with a high surface area and large pores (Figure 7c,d) increased the regions of the sensing reaction and promoted the TEA sensing performance of the sensor.

### 3.5. Ag-Decorated/Loaded Formaldehyde (HCHO) Gas Sensors

**Figure 8:**
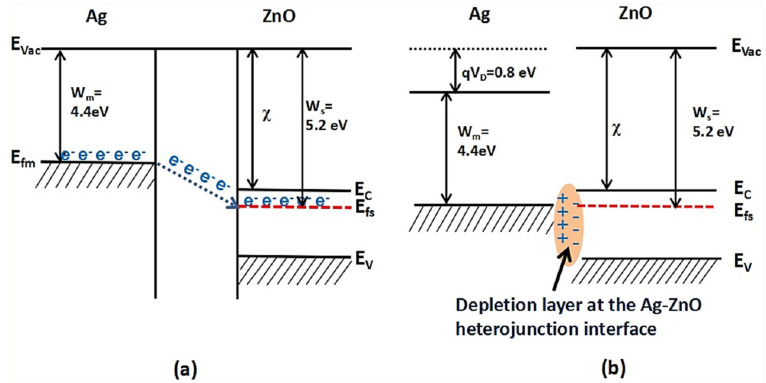
Schematics of the band structures of Ag-loaded ZnO in (**a**) air and (**b**) HCHO. Reprinted from reference [65] with permission from Elsevier.

HCHO is extensively utilized in several industrial applications. Typically, HCHO is a colorless gas with a pungent odor, and it easily evaporates from the products into the indoor air. Many health-related issues, including headaches, nausea, cancer, and mucosal and respiratory irritation, are associated with exposure to HCHO [61,62,63,64]. Therefore, the development of highly sensitive HCHO gas sensors is urgently required to ensure the safety of people. In this regard, Xing et al. [65] prepared highly porous 1–5 at % Ag-loaded ZnO for application in HCHO sensors. In this study, the sensor fabricated using 1 at % Ag-loaded ZnO exhibited the maximum response to HCHO (170.42) at an optimal temperature of 240 °C. Unique hierarchically structured porous Ag-loaded ZnO provided an adequate surface area for interaction between the HCHO molecules and the sensing material. The high response of the optimal gas sensor to HCHO was ascribed to the production of heterojunctions between Ag and ZnO in addition to the catalytic activity of Ag. Schematics of the energy bands of Ag/ZnO in the presence of air and HCHO gas are shown in Figure 8a,b, respectively. Herein, the electrons transferred from Ag to ZnO because the work function of Au (Wm = 4.4 eV) is smaller than that of ZnO (Ws = 5.2 eV), generating an electron-depletion layer because of the increase in the concentration of electrons at the Ag/ZnO interface. When the Ag/ZnO sensor was exposed to air, the electrons released from ZnO were captured by the adsorbed O ions to form O^−^_x_, which showed strong oxidation activity. This process reduced the sensor resistance. Upon exposure to HCHO, the previously adsorbed O^−^_x_ strongly reacted with the HCHO molecules and oxidized HCHO into CO_2_ and H_2_O, thereby decreasing the sensor resistance. This study reveals that the HCHO sensing performance of the sensor is directly influenced by the Ag/ZnO heterojunction and the developed O^−^_x_.

Furthermore, Wang et al. [66] reported the HCHO sensing activity of a two-step solution-processed Ag-loaded sunflower-like hierarchical In_2_O_3_ nanoarchitecture. The observed HCHO sensing performance was attributed to the highly porous and hierarchically designed sunflower-like nanostructure and the catalytic activity of Ag. The exclusive hierarchical 3D sunflower-like nanostructures possess numerous radial nanobranches with irregular surfaces, which offer a larger exposed surface area and additional paths for the exchange of electrons during the entire gas diffusion and surface reaction processes. An appropriate Ag loading amount plays an important role in chemical and electronic sensitization during the detection of HCHO by the Ag-loaded In_2_O_3_ sensor. Chemical sensitization favored gas reactions by dissociating HCHO via a spillover effect, and electronic sensitization substantially enhanced the direct exchange of electrons between In_2_O_3_ and Ag. However, higher Ag loading amount resulted in the conduction of electrons along the metallic Ag NPs irrespective of chemoresistive variations, thereby deteriorating the sensor response.

### 3.6. Ag-Decorated/Loaded Carbon Monoxide (CO) Gas Sensors

CO is among the most hazardous gases as it is invisible, tasteless, odorless, and colorless and thus cannot be detected by human sensory organs [67]. Consequently, numerous efforts have been made to detect CO gas by various materials and strategies. Molybdenum disulfide (MoS_2_) is a distinct graphene-like two-dimensional (2D) layered transition metal dichalcogenide. In addition to a conventional band gap of 1.8 eV, it has a large surface area-to-volume ratio with excellent physical/mechanical properties [68]. Nevertheless, the gradual degradation of MoS_2_ nanosheets occurs under ambient conditions owing to atmospheric oxidation and surface contamination, which may eventually decrease their sensing performances. Thus, to achieve an appropriate CO sensing performance of pure MoS_2_ nanosheets, sensing should be performed under an inert N_2_ atmosphere, significantly restricting their commercial applications. To overcome this limitation, Zhang et al. [69] fabricated a novel ternary Ag-loaded ZnO/MoS_2_ nanocomposite via layer-by-layer self-assembly for the detection of CO gas. 

**Figure 9:**
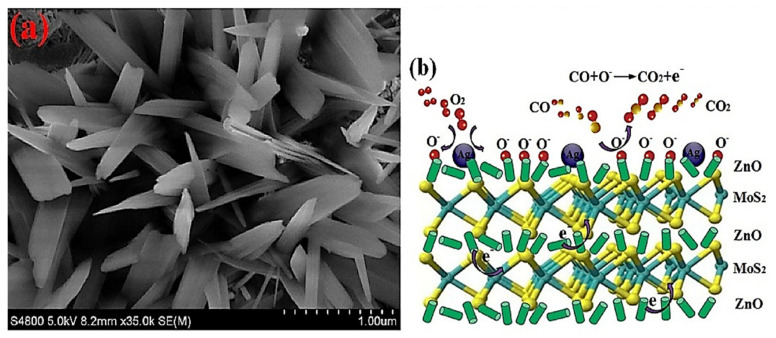
(**a**) SEM image of Ag–ZnO/MoS_2_ and (**b**) schematic of the CO sensing mechanism of Ag–ZnO/MoS_2_. Reprinted from reference [69] with permission from Elsevier.

The presence of ZnO NRs, Ag NPs, and MoS_2_ nanosheets was verified by morphological investigations (the scanning electron microscopy (SEM) image is shown in Figure 9a). The developed nanocomposite sensor demonstrated excellent room temperature CO sensing performance. This confirmed that the existence of MoS_2_ might reduce the operating temperature and energy consumption probably because of the higher specific surface area and better conductivity of MoS_2_ along with the synergistic effect between ZnO and MoS_2_. Ag effectively improved the catalytic activity of the sensor and mobilities of the carriers for the interaction of gas with the surface of MoS_2_ or ZnO by modifying the energy band structure and surface morphology of the sensing material. Furthermore, substantial dynamic sites for CO adsorption might be generated upon Ag loading. After Ag modification, the interactions of CO molecules with O ions dramatically reduced the resistance of the Ag–ZnO/MoS_2_ nanocomposites as compared to that of the ZnO/MoS_2_ film, resulting in a high CO response of the sensor. A schematic of the interactions between the CO molecules and the Ag–ZnO/MoS_2_ sensor is depicted in Figure 9b. Herein, Ag and Pt were employed to improve the CO sensing performance of the ZnO/MoS_2_ composite sensor. The Ag-loaded sensor exhibited the maximum response as compared to that of the Pt-loaded sensor under similar experimental conditions. The higher response of the Ag-loaded sensor was explained with respect to the work function. Upon the introduction of a noble metal into ZnO/MoS_2_, the electrical characteristics of ZnO/MoS_2_ may be influenced by the existence of a Schottky barrier. Therefore, noble metals with lower work functions are preferred to reduce the Schottky barrier and thus improve the response of the corresponding ZnO/MoS_2_ sensor. As Ag has a lower work function (4.26 eV) than that of Pt (5.65 eV), the Ag–ZnO/MoS_2_-based sensor demonstrated higher CO sensing performance.

In another study, Ag-modified flower-like ZnO microspheres were prepared using a combination of solvothermal strategy and an impregnation approach [70]. Dual selective sensing of methane (CH_4_) and CO using 1.5 at % Ag-loaded ZnO by monitoring the working temperature was reported in this study. Ag exhibits high catalytic activity for low-temperature oxidation of CO. However, the low-temperature oxidation of CH_4_ is very difficult to achieve, as CH_4_ is a thermally stable sp^3^-hybridized non-polar molecule. Accordingly, no considerable low-temperature response of the sensor to CH_4_ was observed. In contrast, at higher temperatures, CH_4_ was sufficiently activated by Ag because there was adequate thermal energy to overcome the barrier, thereby increasing the sensor response. Consequently, the sensor fabricated using 1.5 at % Ag-loaded ZnO exhibited temperature-modulated dual selectivity toward CO and CH_4_ at 130 and 200 °C, respectively.

### 3.7. Ag-Decorated/Loaded Ethanol (C_2_H_5_OH) Gas Sensors

**Figure 10:**
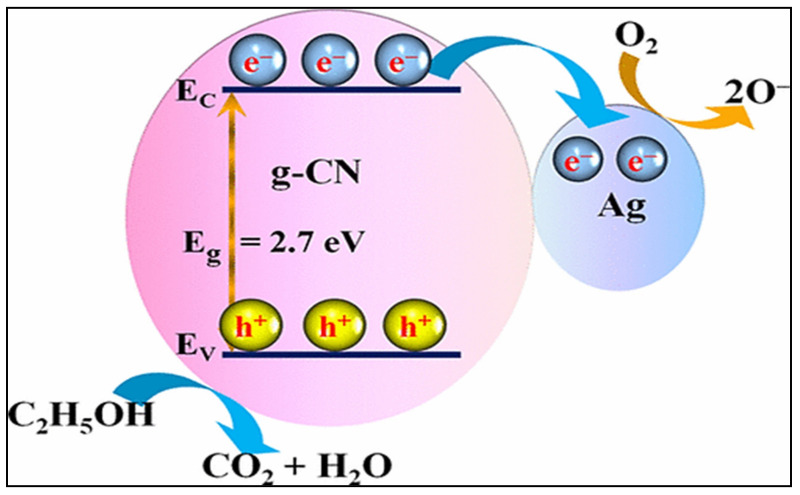
Band diagram of the Ag/g-CN sensor, elucidating the C_2_H_5_OH sensing mechanism of the sensor [72]. Reprinted from https://pubs.acs.org/doi/10.1021/acsomega.7b00479 (accessed on July 14, 2017) with permission from ACS (further permissions related to the material excerpted should be directed to the ACS).

C_2_H_5_OH is one of the most commonly used VOCs. Exposure to C_2_H_5_OH vapors may cause headache, drowsiness, eye irritation, liver damage, and breathing difficulties. Furthermore, the consumption of C_2_H_5_OH is the leading cause of motor vehicle accidents worldwide, and alcohol-impaired driving fatalities account for 31% of the total number of traffic-related deaths in the United States [71]. Thus, the design of low-temperature operating and selective sensors for the detection and monitoring of C_2_H_5_OH is required from the viewpoint of safety. Graphitic carbon nitride (gC_3_N_4_, g-CN) is a non-metallic polymer semiconductor with a 2D layered structure. As a result of its unique physicochemical characteristics, suitable band gap, and outstanding thermal and chemical stabilities, g-CN can be utilized as an active sensing element in gas-sensing applications. In this regard, Tomer et al. [72] fabricated well-ordered mesoporous Ag-decorated meso-CNs using a hard template by nanocasting to detect C_2_H_5_OH at trace levels. The response of cubic meso-CN to C_2_H_5_OH gas was approximately two times that of conventional g-CN. This improvement was caused by the higher meso-CN surface area along with uniform pore channels through which the target gas penetrated the deep portions of the sensor. The response of the 3 wt.% Ag-decorated meso-CNs to C_2_H_5_OH gas was two times that of meso-CN. A schematic of the C_2_H_5_OH sensing mechanism of the Ag/g-CN sensor is shown in Figure 10. In this case, the electrons transferred from g-CN to Ag due to the lower work function of g-CN (4.3 eV) than that of Ag (4.7 eV), thereby creating a potential barrier. This barrier prevented the transfer of electrons through the mesoporous sensor, and the electrons existing on the sensor surface developed dynamic sites for the adsorption of O_2_, thereby increasing the sensor response. Moreover, the loading of Ag NPs strongly accelerated catalytic oxidation and chemical sensitization, which ultimately enhanced the number of dynamic O species on the surface of meso-CN. In fact, the chemical sensitization effect (namely, spillover effect) of Ag NPs improved the rate of adsorption–desorption of molecular O_2_ on the sensor surface and transformed molecular O_2_ into O ions. Furthermore, the excellent catalytic activity of Ag NPs accelerated the decomposition of C_2_H_5_OH gaseous molecules into active radicals, thus enhancing the interactions between the chemisorbed O ions and C_2_H_5_OH molecules. Owing to the transfer of electrons from meso-CN to Ag NPs, a negatively charged layer was produced around the Ag/meso-CN interface. Under a C_2_H_5_OH atmosphere, the electrons returned to the sensing layer via the Ag_2_O/Ag redox reaction, and thus, the areas along the Ag/meso-CN interface became very sensitive to C_2_H_5_OH. 

### 3.8. Ag-Decorated/Loaded Nitrogen Dioxide (NO_2_) Gas Sensors

NO_2_ is a highly poisonous and polluting gas that produces acid rain and photochemical smog [73]. As a result of its highly toxic nature, detection and monitoring systems for NO_2_ are needed. Accordingly, Wang et al. conducted NO_2_ gas-sensing studies employing Ag-loaded mesoporous WO_3_ designed using 3D cubic KIT-6 as a hard template [74]. Herein, Ag at different molar ratios of 0.2, 0.5, and 1.0% was loaded onto mesoporous WO_3_. The sensor developed using 0.5% Ag exhibited a higher response of 44 to 1 ppm NO_2_ at 75 °C because of its larger surface area and diffusion paths. NO_2_ is a polar molecule having a positive charge localized on the N atom and a negative charge over the O atom. The interaction of electrons with Ag repels the O atom and attracts the positively charged N atom. Ag was used as a dynamic catalyst to create more active sites for the detection of NO_2_. Although the addition of Ag may enhance the sensing properties of the material, the loading of Ag at excessively high concentrations can decrease the catalytic performance of the material. When the Ag concentration exceeded 0.5%, the total surface area reduced, and interconnected Ag NPs changed the electron path to be from Ag instead of WO_3_. 

**Figure 11:**
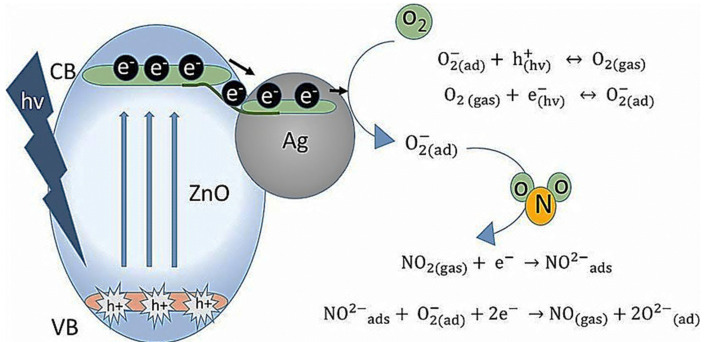
Schematic of electron mobilization and related reactions of the Ag–ZnO sensor in the presence of NO_2_ gas [75]. Reproduced from https://iopscience.iop.org/article/10.1149/2.0141807jss (accessed on 10 April 2018) from IOPScience (This is an open access article distributed under the terms of the Creative Commons Attribution Non-Commercial No Derivatives 4.0 License (CC BY-NC-ND, http://creativecommons.org/licenses/by-nc-nd/4.0/ (accessed on 10 April 2018))).

The advantages of UV-activated sensors are their high stabilities, abilities to detect flammable gases, and low energy consumptions. In a typical UV-based gas-sensing device, the UV wavelength and power intensity determine the energy of the photons arriving at the exposed surface. This energy influences the steady-state surface reactions and plays a significant role in the response curve. Espid et al. [75] explained the NO_2_ gas detection characteristics of Ag-loaded ZnO nanoellipsoids, which were synthesized by a simple coprecipitation method, under UV illumination. When the surface of the gas sensor was irradiated with the photons released from a UV light-emitting diode, the electrons of the sensor hopped from the valence band to the conduction band, leaving holes in the valence band. A schematic of electron mobilization and the related reactions of the Ag-loaded ZnO sensor with NO_2_ gas under UV illumination is depicted in Figure 11. Photogenerated electron/hole pairs supported the direct adsorption of O_2_ molecules. Under an air atmosphere, O_2_ interacted with the excited electrons and adsorbed in the ionic form on the sensor surface. This slightly increased the sensor resistance. Under a NO_2_ atmosphere, the gas molecules reacted with the chemisorbed oxygen ions or directly adsorbed on the sensor surface by accepting electrons. A change in the electron flow stimulated by these reactions modulated the electrical resistivity. Under continuous UV irradiation, when the NO_2_ flow was stopped, the accelerated photons eliminated the NO_2_ species from the sensor surface. The observed enhancement in the NO_2_ detection performance of ZnO nanoellipsoids after Ag loading was associated with the synergistic effects of the semiconductor composite and Ag. Additionally, the improvement of the sensing response in the presence of Ag was linked to the increased electron utilization ratio and the decreased recombination rate of photogenerated electrons and holes due to the trapping of electrons in the Ag NPs during excitation. Similarly, the adsorption ability was directly correlated with the number of unoccupied oxygen sites. When Ag was embedded in the ZnO lattice, O-vacancies formed because of the differences between the charges of Ag^+^ and Zn^2+^ ions, which indicated that additional adsorption sites were created, resulting in a higher sensor response. 

**Figure 12:**
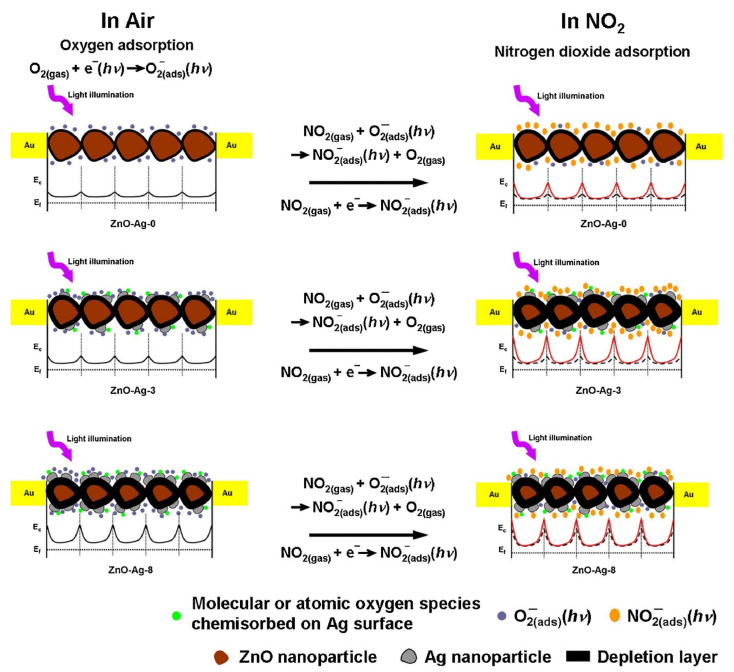
Schematic of the NO_2_ gas detection mechanism of pristine and Ag-loaded ZnO NPs irradiated with light of 365–520 nm wavelengths. Reprinted from reference [76] with permission from Elsevier.

Furthermore, Zhang et al. [76] designed a room-temperature light-assisted NO_2_ gas sensor using Ag-decorated ZnO NPs. The sensor developed using 3 mol.% Ag exhibited the highest NO_2_ response. A schematic of the NO_2_ detection mechanism of pure and Ag-loaded ZnO NPs under various light conditions is shown in Figure 12. Herein, characterization studies revealed the development of a heterojunction between Ag NPs and ZnO NPs. Nevertheless, the improved NO_2_ detection performance of the sensor after Ag loading was ascribed to prominent interactions between Ag NPs and the O vacancies. In this study, Ag NPs and surface oxygen vacancies acted as electron sinks to boost charge separation and catalytic activities of Ag NPs for promoting the adsorption of gas molecules. The effective separation of photogenerated electron–hole pairs due to the interfacial charge transfer between ZnO and Ag and charge-carrier trapping through the surface oxygen vacancies enabled further accumulation of unpaired electrons on the surfaces of ZnO NPs. Moreover, Ag NPs and oxygen vacancies on the surface enhanced the adsorption of gas molecules, and thus, more oxygen molecules adsorbed on the sensor surface. An excess of Ag NPs reversibly acted as charge-carrier recombination centers, leading to negatively charged electrostatic attraction between Ag and the positively charged holes, thereby reducing the photoquantum efficiency. Additionally, sometimes, it is difficult to completely desorb the molecular or atomic oxygen species chemisorbed on the Ag surface through light irradiation at room temperature. In brief, O_2_ molecules play an essential role in the absorption/desorption of NO_2_ molecules on the surfaces of ZnO NPs. Specifically, the exposure degree of O^−^_2(ads)_ (hν) directly stimulates the sensing characteristics, such as response and response/recovery speeds, of light-assisted NO_2_ sensors.

### 3.9. Ag-Decorated/Loaded Methyl Mercaptan (CH_3_SH) Gas Sensors

**Figure 13:**
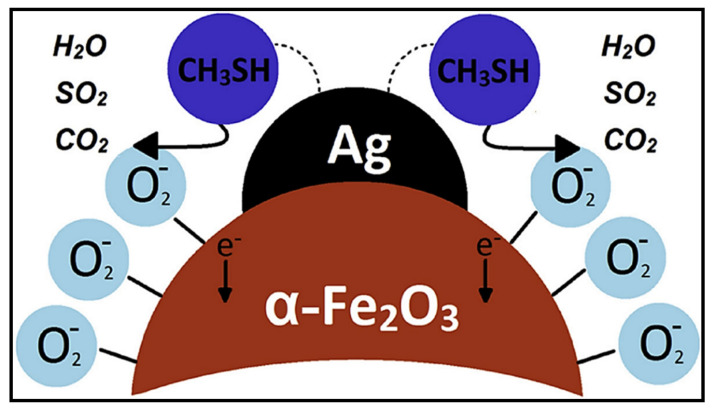
Schematic of the chemical influence of Ag NPs on the sensing mechanism of the α-Fe_2_O_3_ sensor. Reprinted from reference [77] with permission from Elsevier.

CH_3_SH is a gas that is commonly used as an additive to other gases including propane and natural gas. The rotten egg odor of CH_3_SH facilitates the detection of CH_3_SH leakage. The recommended airborne exposure limit for CH_3_SH is 0.5 ppm during an 8 h work shift. Exposure to higher levels of CH_3_SH results in eye and throat irritation, drowsiness, and even bronchitis [77]. Consequently, designing a sensor that can detect CH_3_SH at low levels is highly desirable. Additionally, CH_3_SH gas-sensing properties of SMOs have rarely been reported. In this regard, Garcia et al. [77] constructed a sensor using mesoporous Ag-loaded hematite (α-Fe_2_O_3_) to detect CH_3_SH at room temperature. Herein, the loading of 3 wt.% Ag onto α-Fe_2_O_3_ significantly enhanced the sensor response owing to the deeper electron-depletion layer. Ag loaded onto the surface of α-Fe_2_O_3_ served as a catalyst to accelerate the rate of conversion of O_2_ into ionic O. Therefore, more electrons were trapped, which generated a thicker depletion layer. When the Ag-loaded α-Fe_2_O_3_ sensor was exposed to CH_3_SH, the CH_3_SH molecules favorably chemisorbed due to the strong affinities of thiol groups for metallic Ag. Ag loading accelerated the reaction of CH_3_SH with oxygen ions via a spillover effect (Figure 13). In this case, the deep electron-depletion layer converted into a flat layer, which resulted in a better sensor response. However, when the concentration of Ag exceeded 3 wt.%, the Ag NPs generated a connected network on the surface of α-Fe_2_O_3_, and consequently, the resistance of the sensor reduced. Thus, the adsorption of O_2_ and the gas–O_2_ interaction substantially reduced, causing an inferior response of the sensor to the target gas.

### 3.10. Ag-Decorated/Loaded Xylene (C_8_H_10_) Gas Sensors

**Figure 14:**
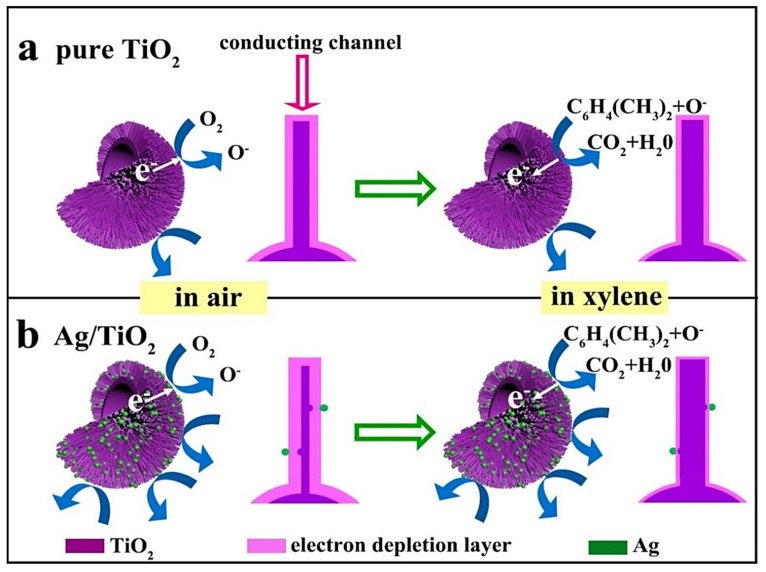
Schematic of the C_8_H_10_ gas-sensing mechanism of (**a**) the pristine and (**b**) Ag-loaded TiO_2_ sensors. Reprinted from reference [80] with permission from Elsevier.

C_8_H_10_ is a VOC that is colorless, odorless, and highly toxic in nature [78,79]. It causes severe damage to the human body, even at very low levels and upon long-term exposure [21]. Prolonged exposure to C_8_H_10_, even at low levels, is very harmful to living organisms; therefore, detection and monitoring of C_8_H_10_ is necessary. In a study reported by Zhang et al. [80], C_8_H_10_ detection properties of 0.2, 0.5, and 1 at % Ag-loaded hedgehog-like TiO_2_ architectures are described. Herein, Ag-loaded hedgehog-like TiO_2_ nanostructures composed of hundreds of 1D NRs were synthesized via a simple hydrothermal process followed by isometric impregnation. Gas-sensing studies showed that the 0.5 at % Ag-loaded TiO_2_ sensor demonstrated the highest C_8_H_10_ response as compared to those of the 0.2 and 1 at % Ag-loaded TiO_2_ sensors. A schematic of the sensing interactions between the C_8_H_10_ gas molecules and Ag/TiO_2_ is depicted in Figure 14. A typical gas-sensing mechanism of n-type SMOs, based on the change in resistance upon the interaction of these SMOs with gases, was used to explain the interactions between C_8_H_10_ and Ag/TiO_2_ [65,66,77]. The C_8_H_10_ responses observed for the pristine and 0.5 at % Ag-loaded TiO_2_ sensors were 3.19 and ≈6.49 at 375 °C, respectively. The sensor response reduced when the Ag loading amount was increased up to 1 at %; this was mainly due to the coverage of the TiO_2_ surface sites by excessive Ag. Interestingly, all the Ag-loaded sensors exhibited fast responses and recovery dynamics; that is, the acquired response and recovery times were ≈5–7 and ≈1–2 s, respectively. The rapid response and recovery times were attributed to the strong electron mobility of the 1D TiO_2_ NRs and the superior porosity of the hedgehog-like Ag-loaded TiO_2_ architecture. The improvement in the C_8_H_10_ sensing characteristics of the TiO_2_ sensor upon Ag loading was ascribed to the catalytic activity of Ag and the development of Ag/TiO_2_ heterojunctions. The catalytic Ag NPs facilitated more and quicker reactions between the adsorbed oxygen ions and the C_8_H_10_ molecules.

### 3.11. Ag-Decorated/Loaded Ammonia (NH_3_) Gas Sensors

NH_3_ is a hazardous gas that is colorless and toxic. It has significant applications in various areas such as compound fertilizers, synthetic fibers, and biofuels. Additionally, exposure to NH_3_ at high levels may result in serious effects, including irritation of the eyes/skin/throat and the respiratory tract, on the human body [81,82]. Karaduman et al. [83] reported the NH_3_-sensing characteristics of Ag-decorated rGO. In this case, when NH_3_ gas molecules adsorbed on the surface of rGO by physisorption, the holes of rGO interacted with the electron-donating NH_3_ gas. Therefore, the degree of delocalization of the conjugated p-electrons of the detecting surface enhanced through the transfer of charge carriers from the adsorbed NH_3_ molecules. This process reduced the concentrations of charge carriers, thereby increasing the electrical resistance of the sensing material. The enhanced NH_3_ sensing response of the Ag-loaded rGO sensor was attributed to the following factors: (i) The functional groups and defects in rGO provided numerous active sites for NH_3_ adsorption, increasing the gas adsorption capacity. (ii) The existence of Ag NPs resulted in the adsorption of more NH_3_ molecules on the rGO surface as NH_3_ molecules strongly bound to the rGO surface. The catalytic characteristics of Ag NPs stimulated the breakdown of NH_3_ into active radicals and enhanced the reaction between chemisorbed O ions and NH_3_ molecules. (iii) The development of an n-AgxOy/p-rGO heterointerface led to a depletion region, which concurrently lowered the concentrations of carriers on both sides. When the sensor was exposed to NH_3_ gas, the electronic conductance of the sensor considerably increased, which improved the sensing response.

**Figure 15:**
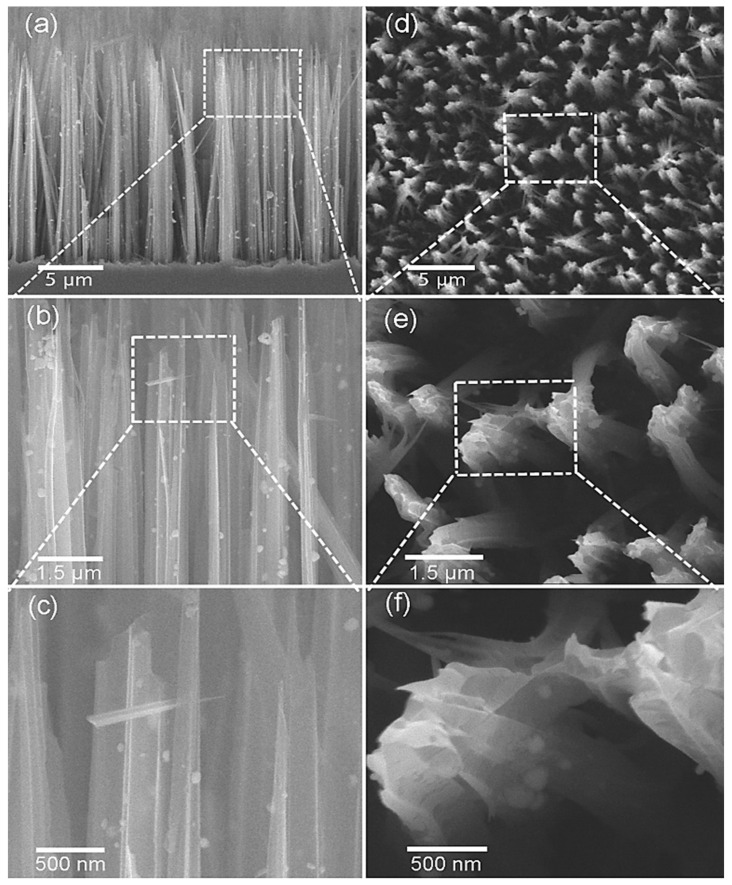
SEM images of Ag-PPy@SiNWs with increasing resolution in front view (**a**–**c**) and planar views (**d**–**f**). Reprinted from reference [84] with permission from Elsevier.

Organic/inorganic hybrid nanocomposites possess unique characteristics owing to the combined properties of polymers and SMOs. Additionally, decoration with noble metals, including Ag, may enhance the unique characteristics of the hybrid materials because of the catalytic activity of Ag. Qin et al. [84] reported the NH_3_-sensing properties of Ag-loaded polypyrrole@Si nanowire (Ppy@SiNW) core–shell structures (Figure 15). 

**Figure 16:**
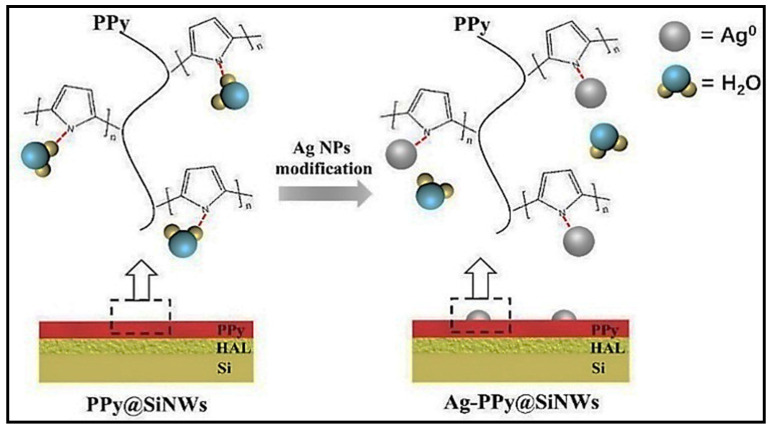
Schematic of the anti-humidity effect caused by Ag NPs. Reprinted from reference [84] with permission from Elsevier.

Herein, electrical transport occurred in both the shell and the nearby core at the Si/PPy interface because of the thin layer of the PPy shell. Moreover, the Ag-loaded sensor exhibited baseline resistance that was higher than that of the pristine sensor, indicating strong electronic sensitization effect of the Ag NPs. NH_3_ sensing measurement was conducted using the Ag-loaded PPy@SiNW sensor under high ambient humidity conditions. In this case, a higher NH_3_ response and weaker humidity interference were noticed following the loading of Ag NPs onto the PPy@SiNW sensor. Actually, the Ag NPs coordinated with –N groups (tertiary N) in the organic chains. Note that the tertiary groups of N are the general coordination sites of the water molecules adsorbed on the polymer surface. Therefore, throughout the polymerization of PPy, Ag^0^ preferably coordinated with –N groups in PPy, which inhibited further adsorption of water on similar –N groups. Simultaneously, the loaded Ag NPs were highly hydrophobic. Thus, no significant water adsorption occurred on the surfaces of the Ag NPs, even at 100% relative humidity. Accordingly, the enhanced anti-humidity interference properties of the Ag-PPy@SiNWs originated from the attenuation of water adsorption on the Ag-loaded PPy shell. The Ag NPs loaded on the PPy chain demonstrated high hydrophobicity (Figure 16), which resulted in nearly no water adsorption on the PPy shells. The minimal adsorption of water molecules was also beneficial for the electronic and chemical effects of Ag NPs. These factors also caused higher response amplitudes at high ambient humidity [84].

### 3.12. Summary of Ag-Decorated Gas Sensors

In the above sections, we have described the effect of Ag decoration on the gas response of resistive-based gas sensors. Table 1 [48,49,50,51,52,54,60,65,66,69,70,72,74,76,77,80,85,86,87,88,89,90,91,92,93,94,95] shows the gas-sensing performances of Ag-loaded SMO-based sensors for various toxic gases. In addition, other researchers [95,96,97,98,99,100,101,102,103,104,105,106,107,108,109] have reported enhanced gas sensing after Ag decoration. Depending on the type of SMO and the synergistic effects between Ag and SMO, the sensing temperature can vary from room temperature to high temperatures. Furthermore, in some cases, higher responses were realized after loading Ag NPs onto the surfaces of SMOs. Overall, in the presence of Ag NPs, not only the sensing temperatures reduced but also higher responses were attained when compared with those of the pure gas sensors, and selectivity improved owing to the catalytic activities of the Ag NPs. 

## 4. Ag-Doped Gas Sensors

**Figure 17:**
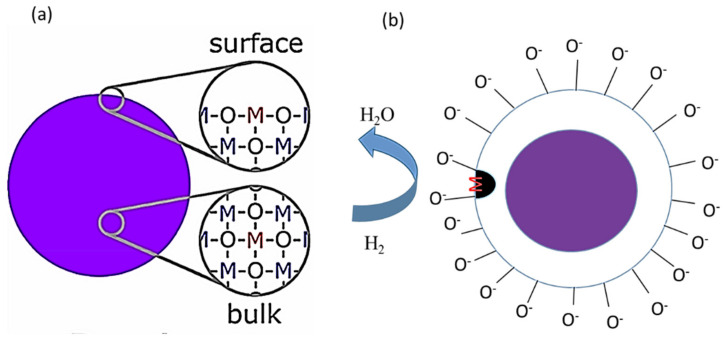
(**a**) Doping of a metal on metal oxide and (**b**) doping effect on the gas interaction with the sensing layer. Reprinted from reference [110] with permission from ACS.

Doped metal oxide is the structure where additives are incorporated in the lattice of host material, as shown in Figure 17a [110], and it can affect the gas response of the doped gas sensor (Figure 17b). In gas-sensing studies, very less attention has been devoted to Ag doping than to Ag loading. 

Ag doping is often performed by incorporating Ag atoms into the SMO lattice. When Ag atoms are homogeneously dispersed in the semiconductor lattice, mobile charge carriers are generated, thereby changing the electrical properties of the gas sensor [111]. An overview of some Ag-doped gas sensors is presented in this section.

Hydrogen sulfide (H_2_S) is a highly toxic gas that is typically produced by the oil industry, natural gas plants, and sewage plants. Exposure to H_2_S at higher levels (namely, 100 ppm) may cause sudden collapse with respiratory loss, and the possibility of fatality is very high [112]. 

**Figure 18:**
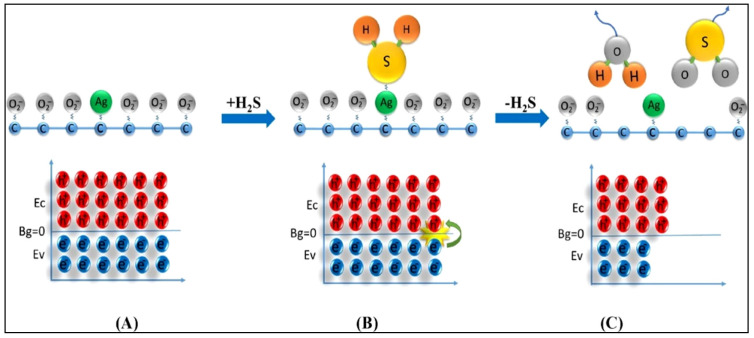
Schematic of H_2_S sensing by Ag-doped graphene. Energy diagram of (**A**) Ag-doped graphene, (**B**) interaction of Ag-doped graphene with H_2_S gas, and (**C**) the formation of SO_2_ and H_2_O. Reprinted from reference [113] with permission from Elsevier.

Ovsianytskyi et al. [113] developed a H_2_S gas sensor using Ag NP-doped graphene. When the Ag-doped graphene sensor was exposed to H_2_S gas, H_2_S adsorbed on Ag rather than on C because Ag is less electronegative than C; the adsorption of H_2_S led to a possible dissociation of H_2_S, and accordingly, SO_2_ and H_2_O were formed by the release of electrons (Figure 18). The released electrons entered graphene and recombined with intrinsic holes. This process decreased the concentrations of charge carriers and increased the resistance of Ag-doped graphene. Furthermore, Kolhe et al. [114] prepared SnO_2_ thin films doped with 1.5, 3.0, and 4.5 mol.% Ag by chemical spray pyrolysis for application in H_2_S sensors. Herein, the 3.0 mol.% Ag-doped SnO_2_ sensor exhibited the maximum H_2_S response. The enhanced response of SnO_2_ thin films toward H_2_S gas upon Ag doping was related to the catalytic activity of Ag or Ag_2_O and the formation of heterojunctions between Ag/SnO_2_ and Ag_2_O/SnO_2_. When the doping concentration of Ag was excessively high, the sensor response decreased because of the decrease in the number of dynamic sites owing to the accumulation of Ag grains. Additionally, Anand et al. [115] synthesized In_2_O_3_ and Ag-doped In_2_O_3_ NPs using a simple coprecipitation technique and utilized them for the detection of C_2_H_5_OH. Ag doping induced defects and vacancies in In_2_O_3_, which improved the number of dynamic sites on the sensor surface and accordingly increased the adsorption of C_2_H_5_OH. Consequently, the width of the depletion layer expanded, and thus, the resistance of the Ag-doped In_2_O_3_ sensor considerably increased. When the sensor was exposed to C_2_H_5_OH gas, the width of the depletion layer substantially reduced, which was a contributing factor to the sensing signal. Moreover, Ag acted as a catalyst and facilitated the sensor response by forming activated species of the chemisorbed oxygen ions, which were subsequently released to the In_2_O_3_ surface and caused the C_2_H_5_OH molecules to rapidly react with the chemisorbed oxygen ions. 

Finally, Ding et al. [116] prepared 0.5, 1.0, and 3.0 mol.% Ag-doped hollow urchin-like In_2_O_3_ spheres via a one-step hydrothermal method for application in NO_2_ sensors. Herein, the sensor fabricated using 1.0 mol.% Ag demonstrated the maximum response to NO_2_ gas at an operating temperature of 62 °C. An excellent response of 190 was obtained to 1 ppm NO_2_ gas at 62 °C, which was almost 23 times that of the pristine In_2_O_3_ sensor. The improvement in the NO_2_ sensing response of the In_2_O_3_ sensor after Ag doping was ascribed to the chemical sensitization effect of Ag, which acted as a dynamic catalyst and produced more active sites on the sensor surface. The generated active sites caused the direct adsorption of NO_2_, which finally led to a higher NO_2_ response. When the Ag doping concentration was low, the catalytic effect was inadequate to make the entire surface of the sensor available for the adsorption of NO_2_ gas. Nevertheless, when 3.0 mol% Ag was doped, the sensor response decreased. Ag at higher doping concentrations covered the active sites on the sensor surface, which prevented the sensor from responding to the NO_2_ gas. Furthermore, the surface area of the optimal sensor was 100.6 m^2^/g, which also contributed to the improved gas response. 

Table 2 presents the sensing properties of Ag-doped SMO-based gas-sensing devices [92,93,94,95,96,97,98,99,100,101,102,103,104,105,106,107]. Different gases at various temperatures can be detected by doping Ag into the pristine sensing device, which suggests the promising role of Ag as a noble metal dopant in the detection of toxic gases.

## 5. Conclusions and Perspectives

In this review, we have examined the promising effects of Ag addition on the detection performances of chemiresistive gas sensors. This review demonstrated that several toxic gases and VOCs can be efficiently detected by introducing Ag into SMOs and corresponding composite-based chemiresistive sensors. The gas-sensing performances of chemiresistive sensors, such as SMOs and related composites, can be enhanced by Ag doping owing to the catalytic activity and electronic/chemical sensitization effects of Ag. As an effective catalyst, Ag can attract abundant O_2_ molecules from the air and transfer them to the surfaces of SMOs, accordingly promoting the capture of electrons from SMOs by O_2_ molecules. Moreover, the higher electrical conductivity of Ag NPs facilitated rapid electron transfer and thereby improved the sensor response. Therefore, Ag may be an excellent choice as a sensitizer to improve the sensing performances of chemiresistive sensors as it offers additional active adsorption sites and charge transfer pathways to enhance surface reactions. Generally, the introduction of Ag in an optimal amount into the sensing material can lead to the best sensing properties, and a bell-shaped relationship typically exists between the sensor response and the addition amount of Ag [129]. Due to the low cost of Ag than that of other noble metals, the incorporation of Ag into gas sensors is a highly promising strategy to not only reduce the overall price of these sensors but also enhance their sensing properties. 

Although different approaches have been introduced for the synthesis of Ag-based chemiresistive sensors, prospects for the development of Ag-based chemiresistive sensors are still considerable. Nevertheless, to date, the effect of Ag particle size on the gas sensor response has not been systematically investigated, and it should be realized in future efforts related to the decoration of Ag on the surfaces of SMOs and related composites. Additionally, the effects of annealing temperature on the final responses of Ag-decorated gas sensors have not been examined to date, and it may constitute another exciting area of research. In fact, less efforts have been dedicated toward studying Ag doping than those toward studying Ag decoration on the surfaces of SMOs for the detection of gases. Particularly, further Ag doping studies should be conducted in the future. Furthermore, selectivity toward a target gas remains a major concern for Ag-based SMOs and related composite-based resistive gas sensors. Additionally, most of the reported Ag-based chemiresistive sensors operate at higher temperatures, which hinders their commercialization. Therefore, the development of novel functional nanomaterials with superior characteristics is required to address this issue. Efforts should be made to develop room-temperature sensors to reduce energy consumption with enhanced sensitivity. For example, UV irradiation [130], bimetallic decoration [131], use of quantum dots [132] or their combinations [133] are among different strategies that can reduce the sensing temperature even to room temperature. In addition, a fluctuation-enhanced sensing (FSA) approach can be used to improve the sensitivity of the sensors [134,135,136,137]. 

Moreover, understanding the plausible detection mechanism of a specific target gas is a challenge in the field of chemiresistive sensors. Thus, to gain a deeper understanding of the basic sensing principles and respective surface adsorption/desorption kinetics between the target gas and sensing materials, appropriate technology or reasonable models need to be established. More importantly, all reported Ag-based chemiresistive sensors are laboratory-made and probably contain instrumental errors. Thus, further studies are necessary to enhance the robustness of the data, and the respective efforts must be dedicated to the commercialization of these devices in practice. 

## Figures and Tables

**Table 1:** Gas-sensing properties of Ag-decorated SMO-based gas sensors for different gases.

Sensing Materials	Gas Type	GC (ppm)	T (°C)	Response	Ref.
Ag-loaded SnO_2_ hollow NFs	CH_3_COCH_3_	50	160	42	[48]
Ag-decorated TiO_2_ NRs	CH_3_COCH_3_	3.8	200	7.31 (ΔI)/I_0_	[49]
Ag-loaded BiFe_2_O_4_ NPs	Cl_2_	10	240	72.62	[50]
Ag-decorated ZnO/G nanocomposite	C_2_H_2_	100	150	21.2	[51]
5 wt.% Ag-loaded ZnO NPs	C_2_H_2_	100	200	≈80%	[52]
Ag-loaded ZnO NRs	C_2_H_2_	1000	200	27.2	[54]
Ag-loaded ZnO NPs	TEA	100	183.5	6043	[60]
Ag-loaded Porous ZnO NPs	HCHO	100	240	180.4	[65]
Ag-loaded In_2_O_3_ sunflower structure	HCHO	20	240	11.3	[66]
Ag-loaded ZnO/MoS_2_	CO	100	25	6	[69]
Ag-loaded ZnO NPs	CO	100	130	24.17	[70]
Ag-loaded ZnO NPs	CH_4_	5000	200	20.15	[70]
Ag/g-CN	C_2_H_5_OH	50	40	1.3	[72]
Ag-loaded mesoporous WO_3_	NO_2_	1	75	40	[74]
Ag-loaded ZnO NPs	NO_2_	5	25 (UV light)	1.545	[76]
Ag-loaded Fe_2_O_3_	CH_3_SH	80	25	72%	[77]
Ag-loaded TiO_2_ hedgehog-like architecture	C_8_H_10_	100	375	6.9	[80]
Ag-loaded SnO_2_ yolk-shell nanostructures	H_2_S	5	350	614.9	[85]
Ag-loaded ZnO/rGO	C_2_H_2_	1000	200	33	[86]
Ag-loaded ZnO NRs	C_2_H_2_	100	250	255	[87]
3.5 wt.% Ag-decorated ZnO	C_2_H_5_OH	50	325	32.5	[88]
Ag-decorated ZnO/Graphene nanocomposite	CH_3_COCH_3_	1000	175	71	[89]
Ag-loaded 3D porous flower-like ZnO NPs	C_2_H_5_OH	200	300	268	[90]
Ag-loaded In_2_O_3_ NPs	HCHO	50	210	156.9	[91]
Ag-loaded SnO_2_-rGO nanocomposite	C_2_H_2_	500	90	26 (%)	[92]
Ag-loaded LaFeO_3_ NPs	C_7_H_8_	5	215	24	[93]
Ag-loaded MoO_3_ nanobelts	TEA	100	240	26.58	[94]
Ag-decorated TiO_2_ QDs	NH_3_	20	25	25.1	[95]

T = operating temperature of the sensor, GC = gas concentration, and ppm = parts per million

**Table 2:** Gas-sensing properties of Ag-doped SMO-based gas sensors.

Sensing Materials	Gas Type	GC (ppm)	T (°C)	Response	Ref.
Ag-doped graphene	H_2_S	50	25	140%	[113]
Ag-doped SnO_2_	H_2_S	450	200	1.32	[114]
3% Ag-doped In_2_O_3_	C_2_H_5_OH	1000	300	175	[115]
Ag-doped In_2_O_3_ NPs	NO_2_	1	62	190.1	[116]
Ag-doped ZnO nanoneedles	CH_3_COCH_3_	100	370	19	[117]
Ag-doped ZnO nanoellipsoids	CH_3_OH	200	370	15.8	[118]
Ag-doped LaFeO_3_ NPs	HCHO	100	230	20	[119]
Ag-doped Fe_2_O_3_ NPs	H_2_S	100	400	220	[120]
Ag-doped CaCu_3_Ti_4_O_12_ NPs	H_2_S	10	250	110	[121]
Ag-doped In_2_O_3_ NPs	C_2_H_5_OH	150	100	100	[122]
Ag-doped Zn_2_SnO_4_/SnO_2_ hollow NPs	HCHO	140	50	62.2	[123]
Ag-doped SnO_2_ NPs	H_2_	50	300	25	[124]
Ag-doped ZnO NWs	C_2_H_5_OH	1	300	203%	[125]
Ag-doped In_2_O_3_ NFs	HCHO	600	120	130	[126]
Ag-doped WO_3_	C_2_H_5_OH	100	300	65	[127]
Ag-decorated/Ag-doped ZnO columnar films	C_2_H_5_OH	100	250	145	[128]

T = operating temperature of the sensor, GC = gas concentration, and ppm = parts per million

## Data Availability

Not applicable.
